# Hormone replacement in survivors of childhood cancer and brain tumors: safety and controversies

**DOI:** 10.1530/EC-22-0382

**Published:** 2022-12-14

**Authors:** Ichelle Maa van Roessel, Boudewijn Bakker, Hanneke M van Santen, Wassim Chemaitilly

**Affiliations:** 1 Department of Pediatric Endocrinology, Wilhelmina Children's Hospital, University Medical Center Utrecht, AB Utrecht, The Netherlands; 2 Princess Máxima Center for Pediatric Oncology, AB Utrecht, The Netherlands; 3 Division of Pediatric Endocrinology, UPMC Children’s Hospitalof Pittsburgh, Pittsburgh, Pennsylvania, USA

**Keywords:** childhood cancer, childhood brain tumors, growth hormone, hypothyroidism, hypogonadism

## Abstract

Childhood cancer survivors are at risk for developing endocrine disorders, including deficits in growth hormone, thyroid hormone and sex hormones. The influence these hormones have on cell growth and metabolism has raised concerns regarding the safety of their use as treatments in survivors of childhood cancer and brain tumors. This article offers a summary of current knowledge, controversies and areas for future research pertaining to this area.

## Introduction

Endocrine disorders affect up to 60% of childhood cancer survivors (CCS) (1). Cancer, brain tumors and their treatments have been associated with a higher risk of deficiencies in growth hormone (GH), thyroid hormone and sex hormones. The influence these hormones have on cell growth and metabolism has raised concerns regarding the safety of their use as medications in CCS. Clinical practice guidelines have generally referred medical providers to existing endocrinology guidelines that are not specific to CCS while emphasizing the need to take into consideration potential risks, including tumor recurrence and secondary neoplasia, that are specific to this population (2). Furthermore, recommendations have varied according to the regional or national bodies that have formulated them (2, 3, 4). For these reasons, gaps in knowledge of the true risk conferred by hormone replacement therapy in CCS continue to represent a challenge, as best illustrated by the controversy surrounding the use of GH in this population (2, 3, 4, 5, 6). This review offers a summary of current evidence pertaining to hormone replacement using GH, thyroid hormone and sex hormones in CCS and their potential association with tumor progression or recurrence as well as the risk for subsequent neoplasms in order to facilitate patient counseling and treatment decisions. Medical providers should be aware that CCS are also at risk for adverse health outcomes because of delayed or inaccurate diagnoses, suboptimal treatment and failure to take into consideration the interplay between co-occurring deficits including hypothyroidism and adrenal insufficiency; the discussion of these challenges is however outside the scope of this review.

## Safety of growth hormone replacement

GH deficiency is among the most frequently reported endocrine complications in children treated for brain tumors and CCS with a history of tumor, surgery, or radiotherapy involving the hypothalamic–pituitary region (7, 8, 9). The prevalence of GH deficiency was reported at 12.5% in a heterogeneous group of childhood brain tumor survivors (10); its 4-year cumulative incidence exceeded 90% in children treated for medulloblastoma (9). The risk of GH deficiency increases in a both time- and dose-dependent manner in relation to radiotherapy: the risk increases at higher doses of irradiation and with the duration of follow-up (11). CCS at risk for GH deficiency other than brain tumor survivors include survivors of acute lymphoblastic leukemia with a history of cranial irradiation (12), those treated with total body irradiation prior to hematopoietic stem cell transplantation and survivors of non-brain solid tumors of the head whose treatment included radiotherapy (1). Data supporting an association between GH deficiency and conventional, including intrathecal, chemotherapy are limited (8, 13). In more recent years, tyrosine kinase inhibitors (TKIs) and immune checkpoint inhibitors have been identified as possibly causing GH deficiency (14). The diagnosis of GH deficiency in CCS follows a similar approach than in non-CCS patients, with some adjustments in terms of dynamic test agent choice, as recently summarized by the Endocrine Society Guidelines (2). The treatment of GH deficiency relies on the use of human recombinant GH (hGH). This allows affected children to improve their linear growth and achieve better final height outcomes with added benefits on bone health, lean body mass and quality of life that have motivated the extension of treatment indications to adults with GH deficiency in more recent years (15, 16). The treatment approach of GH-deficient CCS is similar to that used in non-CCS patients (2). Nevertheless,* in-vitro* and* in-vivo* proliferative and pro-mitogenic properties of GH and insulin-like growth factor-1, which is produced following stimulation by GH, have raised concerns regarding potential associations between hGH use and the risk for tumor recurrence or subsequent malignancy in treated childhood cancer and brain tumor survivors. This concern has prompted the publication of over the past three decades many reports and reviews on the topic including a recent consensus statement from the GH Research Society (6, 17).

The first reports of possible association between treatment with GH and cancer risk were contemporary to the use of human-derived (cadaveric) pituitary GH; these included concerns for a higher risk of *de-novo* leukemia (18) and mortality from cancer (19). These findings were not replicated after the introduction of recombinant (deoxyribonucleic acid derived) hGH (20). Safety concerns were nevertheless reignited after the Childhood Cancer Survivor Study (CCSS) reported independent and statistically significant associations between GH replacement and a higher risk of secondary neoplasms in CCS (21, 22), followed by a manuscript from the French contributors to the Safety and Appropriateness of Growth Hormone Treatments in Europe (SAGhE) study reporting a higher-than-expected rate of mortality from bone tumors in individuals treated with GH during childhood for idiopathic GH deficiency and other non-GH deficiency-related indications (CCS were excluded from this particular report) (23).

The CCSS is a large North American multicenter cohort, with participants contributing primarily through self-report to assessments of healthcare utilization and various health outcomes. Three reports from this cohort have attempted to specifically investigate associations between a history of treatment with GH and mortality from cancer, tumor recurrence and /or secondary neoplasia (21, 22, 24). The first two reports elicited a statistically significant and independent association between a history of treatment with GH and the occurrence of secondary neoplasms, primarily due to higher-than-expected rates of meningioma, a tumor with a known association with cranial radiotherapy; neither report found a significant association between treatment with GH and death from cancer or primary tumor recurrence (21, 22). A third, more recent, report specifically investigating the risk for secondary central nervous system (CNS) tumors did not find a significant association between a history of treatment with GH and the occurrence of these lesions (24). The lack of association between treatment with hGH and tumor recurrence, secondary neoplasia and/or increased mortality from cancer was reported by other investigators in other centers, including a recent meta-analysis published by the Endocrine Society (4, 25). The SAGhE cohort is a multicenter European study that utilizes data from national, regional or local registries to assess adverse outcomes associated with hGH (19). Several SAGhE reports have investigated tumor-related outcomes in patients treated with hGH (19, 23, 26, 27). The primary limitation of the SAGhE cohort data is the reliance on comparison of adverse health outcomes and mortality among patients treated with hGH to data from the general population (27). The absence of a non-GH-treated CCS control group curtails investigator ability to identify a specific effect of hGH, beyond risks that are already conferred by the primary cancer or tumor and their treatments (6). Nevertheless, a recent report from this cohort reassuringly showed that the occurrence of meningioma in CCS did not correlate with the dose or duration of treatment with hGH (26).

Benefits of treatment with hGH have been deemed to outweigh potential risks in GH-deficient children who achieve remission from cancer or CNS tumors (6). However, areas of uncertainty remain including whether treatment with hGH should be delayed by 1 year after completion of tumor/cancer therapy (28), whether the dosing of hGH should be different in this population and whether the overall reassuring safety data are truly applicable to GH-deficient children with cancer predisposing syndromes (6). The safety of hGH in patients who require maintenance treatment with targeted chemotherapy for disease control, such as children on TKI, and the use of long-acting hGH preparations are additional areas of uncertainty given the lack of long-term data (6). In adult CCS with GH deficiency, the benefit of hGH is less compelling than in children given that final height, a major outcome that is controlled by GH, has already been attained. The GH Research Society consensus statement allows consideration of hGH treatment in GH-deficient adult survivors who achieve complete remission after careful and individualized analysis of the risks and benefits, with additional caution in patients treated for certain solid tumors (breast, prostate, colon and liver) deemed at higher risk of recurrence (6). The benefits of hGH in adult CCS and those who survive childhood CNS tumors, and how these compare to the yield from other approaches targeting body composition, bone health, lipids and quality of life, thus remain active areas for investigation.

## Safety of thyroid hormone replacement

Hypothyroidism is among the most common endocrine complications after treatment for childhood cancer and brain tumors. CCS have an increased risk for both primary and central hypothyroidism (29). Primary hypothyroidism is defined by decreased thyroid function due to the injury of the thyroid gland itself rather than the hypothalamic–pituitary axis that controls it. Primary hypothyroidism has been reported in 14.7% of CCS and may occur after thyroid surgery or radiation to the neck (30). The rates of primary hypothyroidism were 4%, 7%, and 13% after mean thyroid doses of 10, 20, and 30 Gy, respectively (31). Other treatments known to cause primary hypothyroidism include ^131^I-MIBG, tyrosine kinase and immune checkpoint inhibitors (32). Central hypothyroidism (or thyroid-stimulating hormone (TSH) deficiency) occurs because of the injury of the hypothalamic–pituitary axis due to tumor growth, surgery or radiotherapy. It is frequently reported in children who develop tumors in the sellar or suprasellar region such as craniopharyngioma (33) or optic pathway/hypothalamic glioma or after cranial irradiation including the hypothalamus or pituitary in the radiation field such as for the treatment of medulloblastoma (10). The prevalence of central hypothyroidism in CCSS has been reported at 5.5% in the overall population, 11% among those treated with cranial irradiation and 55% among those with hypothalamic–pituitary tumor involvement (8). At-risk CCS should be periodically screened for hypothyroidism and treated when appropriate due to the health consequences of this condition (5). Untreated childhood hypothyroidism may affect all organ systems and result in decreased energy, neurocognitive impairment, compromised linear growth and delayed puberty. Long-term, primary hypothyroidism has been associated with frailty, dyslipidemia and impaired physical quality of life in CCS (30). Given the trophic effect of TSH on epithelial thyroid cells, supraphysiologic doses of thyroid hormones, high enough to suppress TSH values (TSH suppressive therapy), are used in order to limit the growth of potential residual thyroid cancer cells in the postoperative management of patients with differentiated thyroid carcinoma (34). It is unclear if chronic elevations in TSH from primary hypothyroidism could increase the risk for secondary thyroid neoplasia in at-risk CCS; this is an active area for research (30). In CCS with central hypothyroidism, declining FT4 values have been associated with weight gain, dyslipidemia and glucose disorders (35).

Concerns surrounding the safety of thyroid hormone replacement in CCS have been minimal compared to treatment with hGH. Treatment of hypothyroidism in CCS is generally considered less controversial than that of GH deficiency and hypogonadism. Nevertheless, thyroid hormones are known to play a role in oncogenic pathways involving cell growth, inhibition of apoptosis and stimulation of angiogenesis (36). Thyroid hormones have recently been shown to potentially interact with cancer stem cells, the latter representing a reservoir of cancer, including metastatic cells; controlling intracellular thyroid hormone availability was suggested as a possible tool for reigning in cancer stem cell differentiation in the colon (37). Except for differentiated thyroid cancer, where TSH suppression is used as a treatment strategy as discussed above, there are currently no studies that have investigated thyroid function or thyroid hormone concentration as risk or prognostic factor in childhood cancer. In non-CCS adults, hyperthyroidism has been associated with increased cancer risk, especially lung, prostate, breast, ovarian and thyroid cancer (36, 38). Correlations have also been reported between serum thyroid hormone levels in the upper third tertile and risk for breast, prostate and lung cancer (39, 40). In addition, hyperthyroidism and toxic nodular goiter were reported to influence cancer prognosis (41). Conversely, higher TSH concentrations, as a reflection of hypothyroidism, have been negatively correlated with prostate and breast cancer risk (42, 43). No studies have reported an effect of treating hypothyroidism with thyroid hormones with the aim of achieving euthyroidism on cancer prognosis or recurrence.

In summary, there is no current evidence to support that restoring euthyroidism will have a negative effect on cancer-related health outcomes in CCS. The negative effects of hypothyroidism on metabolism, growth and cardiovascular health in CCS (30, 34) outweigh hypothetical risks that maybe extrapolated from epidemiological studies of non-CCS adults. Studies are needed to specifically investigate associations between thyroid function and childhood cancer prognosis.

## Safety of sex hormone replacement

Hypogonadism (sex hormone deficit) is among the most prevalent complications of childhood cancer, brain tumors and their treatments (44). The management of hypogonadism relies on sex hormone replacement to enable pubertal development during adolescence and help prevent the negative consequences of low testosterone or estrogen levels such as fatigue, decreased muscle strength and decreased bone density later in life (45). Premature ovarian insufficiency (POI) is defined by decreased sex hormone production and impaired fertility due to ovarian injury by cancer or, more commonly, its treatments. The overall cumulative risk of POI in CCS is 11%, and the risk is the highest following ovarian exposure to radiotherapy and treatment with alkylating agents (46). Male CCS are at risk for impaired testosterone production after Leydig cell failure (LCF) following testicular exposure to high-dose radiotherapy and/or alkylating agents. The prevalence of LCF has been reported at 7% in a large cohort of CCS (47). Hypogonadotropic hypogonadism is defined as sex hormone deficit occurring because of hypothalamic–pituitary injury (44). Up to 4% of children with a brain tumor, not including craniopharyngioma, developed hypogonadotropic hypogonadism after exposure to cranial radiation (44). The prevalence of hypogonadotropic hypogonadism in a large cohort of adult CCS was estimated at 10.6% (8). The non-treatment of hypogonadism has been associated with worse general health outcomes in CCS, including cardiovascular risk, decreased bone density and frail health (7, 8, 46, 47).

Sex steroids have been reported to promote cell growth and proliferation* in vitro*. Two of the most common cancer types, breast and prostate cancer, may be influenced by sex hormones (48, 49, 50). This raises the question whether cancer or brain tumors diagnosed during childhood may be influenced by sex steroids, during treatment or after achieving complete remission, and whether sex steroid treatment in CCS increases the risk for secondary malignancies. Several* in-vitro* studies have showed the presence of estrogen, progesterone and/or testosterone receptors on brain tumor tissue. Receptor activity of testosterone and estrogen showed proliferative effects on glia cells mediated via activated androgen receptors and estrogen-alfa receptors, whereas estrogen-beta receptors seemed to have a protective effect against glia cell growth (51). In humans, the relation between brain tumor incidence and female sex steroids was assessed in two meta-analyses. Both did not show higher incidence of glioma in women taking oral contraceptives or hormone replacement therapy, reporting an even lower incidence of glioma in women on sex hormone therapy compared to women not taking these (52, 53). Fewer studies are available in men. A case–control study reported that adults with brain tumors had higher prenatal testosterone levels and lower estrogen levels, possibly explaining that glioma is more prevalent in males than in females (54). Limitations of this study included a small population sample size and the use of the second-to-fourth-digit ratio *in utero* as reflection of prenatal estrogen and testosterone exposure (55).

Observations of disease progression and relapse during puberty of low-grade glioma (LGG) have raised questions concerning the possible contribution of sex hormones to tumor growth in this setting. However, this phenomenon has yet to be confirmed, as literature on this subject is scarce. One study in Canada showed higher incidence of relapse and progression of LGG during pubertal period, but the full manuscript was never published (56). Growth of an intracranial germ cell tumor (iGCT) was reported in a case report of an adolescent treated with human chorionic gonadotropin injections to induce puberty (57). Another case report showed sudden progression of an iGCT after start of puberty, while the tumor had not shown any growth over the previous 7 years (58). A case series on three patients with craniopharyngioma who were treated with estrogen showed tumor enlargement after start of treatment (59). These clinical observations and case reports hint at a possible correlation between start of puberty in children and brain tumor growth. In adults, there is no convincing evidence that incidence or progression of brain tumors is directly influenced by sex hormones. This may indicate that other factors during puberty may be at play (60, 61). In terms of risk for secondary solid tumors, concerns have been raised regarding the risk conferred by treatment with estrogen on breast cancer in at-risk CCS, especially women with a history of chest irradiation (62). Although decreased risk for breast cancer was found for women entering early menopause (63) and in CCS with radiation therapy on the ovaries and use of alkylating agents (64), there is currently no evidence that replacing estrogen deficiency in females at reproductive age increases the risk for breast cancer or other secondary malignancies above levels observed in women without POI (65, 66). Future large cohort studies are needed to evaluate the potential relationship between timing of puberty and/or treatment with sex hormone replacement on one hand and childhood brain tumor relapse or progression and sex hormone-dependent tumor growth on the other hand.

## Conclusion

The safety of treatment with hGH has been investigated in numerous reports pertaining childhood cancer and CNS tumor survivors, with reassuring data regarding the use at substitutive doses in children with proven GH deficiency who have not attained final height and after they achieve disease remission. Areas of uncertainty regarding the risks associated with hGH pertain to management (when to initiate hGH and safe doses of hGH) and to difficulties in the assessment of added risks for adverse tumor outcomes conferred by hGH in children who require maintenance chemotherapy, in those with cancer predisposing syndromes and, overall, in adult survivors of childhood cancer and brain tumors. To the best of our knowledge, despite the proliferative potential of thyroid hormones and sex steroids, there is currently no evidence supporting worse cancer or tumor outcomes when CCS with established deficiencies in these hormones are treated at substitutive doses.

## Declaration of interest

The authors have no conflicts to declare.

## Funding

This work did not receive any specific grant from any funding agency in the public, commercial or not-for-profit sector.
